# *Rhodosporidium toruloides* - A potential red yeast chassis for lipids and beyond

**DOI:** 10.1093/femsyr/foaa038

**Published:** 2020-07-02

**Authors:** Zhiqiang Wen, Sufang Zhang, Chuks Kenneth Odoh, Mingjie Jin, Zongbao K Zhao

**Affiliations:** School of Environmental and Biological Engineering, Nanjing University of Science & Technology, 200 Xiaolingwei St, Nanjing 210094, China; Laboratory of Biotechnology, Dalian Institute of Chemical Physics, CAS, 457 Zhongshan Rd, Dalian 116023, China; Laboratory of Biotechnology, Dalian Institute of Chemical Physics, CAS, 457 Zhongshan Rd, Dalian 116023, China; School of Environmental and Biological Engineering, Nanjing University of Science & Technology, 200 Xiaolingwei St, Nanjing 210094, China; Laboratory of Biotechnology, Dalian Institute of Chemical Physics, CAS, 457 Zhongshan Rd, Dalian 116023, China; Dalian Key Laboratory of Energy Biotechnology, Dalian Institute of Chemical Physics, CAS, 457 Zhongshan Rd, Dalian 116023, China

**Keywords:** chassis organism, genetic modification, microbial lipids, multi-omic analysis, *Rhodosporidium toruloides*

## Abstract

The red yeast *Rhodosporidium toruloides* naturally produces microbial lipids and carotenoids. In the past decade or so, many studies demonstrated *R. toruloides* as a promising platform for lipid production owing to its diverse substrate appetites, robust stress resistance and other favorable features. Also, significant progresses have been made in genome sequencing, multi-omic analysis and genome-scale modeling, thus illuminating the molecular basis behind its physiology, metabolism and response to environmental stresses. At the same time, genetic parts and tools are continuously being developed to manipulate this distinctive organism. Engineered *R. toruloides* strains are emerging for enhanced production of conventional lipids, functional lipids as well as other interesting metabolites. This review updates those progresses and highlights future directions for advanced biotechnological applications.

## Teaser

A review updates research progresses on the red yeast *Rhodosporidium toruloides*and highlights future engineering directions.

## INTRODUCTION

Lipids such as triacylglycerols and fatty acid derivatives are formerly sourced from plants and animal fats. When used as commodity, lipids are important feedstocks for oleochemicals and drop-in biofuels (d'Espaux *et al*. 2015; Jin *et al*. 2015; Leong *et al*. 2018). Compared with extraction from oil crops and plants, microbial production of lipids has many advantages including short production cycle, tailored processes and better accessibility to structural diversity. Therefore, intensive efforts have been made to design advanced strains for microbial production of lipid-based biofuels and chemicals (Yu *et al*. 2018; Zhou, Kerkhoven and Nielsen 2018; Yan and Pfleger 2020). In particular, *Saccharomyces cerevisiae* and *Yarrowia lipolytica* have been engineered to produce diverse oleochemicals. However, these yeasts remain ineffective in xylose utilization and inhibitor resistance, hence preventing large-scale production from lignocellulosic biomass (Ledesma-Amaro and Nicaud 2016; Spagnuolo *et al*. 2018).

Many natural oleaginous microorganisms intracellularly accumulate lipids under nutrient limitation conditions. In the past two decades, the red yeast *R. toruloides* has been used for lipid production from diverse feedstocks and with different bioprocess strategies (Li, Zhao and Bai 2007; Lin *et al*. 2010; Zhao *et al*. 2011; Xu *et al*. 2012; Shen *et al*. 2013; Dias *et al*. 2015; Fei *et al*. 2016). A successful pilot-scale process was demonstrated at 1000-L scale using sugarcane juice as substrate by *R. toruloides* DEBB 5533 for microbial lipid production, and a preliminary analysis showed its economic competitiveness with soybean oil in terms of biodiesel production (Soccol *et al*. 2017). Meanwhile, an early systems study on *R. toruloides* pioneered by Prof. Zhao's group at Dalian Institute of Chemical Physics has paved the way for further engineering of advanced strains (Zhu *et al*. 2012). Thus far, genetic tools including the CRISPR-Cas9 technology have been developed. Engineered *R. toruloides* strains have emerged for improved production profiles or increased product portfolios. Very recently, brief reviews on *R. toruloides* have also been published (Xu and Liu 2017; Park, Nicaud and Ledesma-Amaro 2018; Osorio-González *et al*. 2019a; Saini *et al*. 2020). Here, we present an extensive summary of research progresses on *R. toruloides* and offer discussions on the bottlenecks and perspectives to explore *R. toruloides* as a distinctive chassis for further engineering.

### General background of *R. toruloides*

*Rhodosporidium toruloides* is a red heterothallic, dimorphism yeast, first isolated in 1922 from the air in Dalian, China and named as *Torula rubescen* (Banno 1967). It can exist either in the yeast form or as a mycelial form. In nature, *R. toruloides* is also found in pine wood pulp, soil, seawater, acid sewage and plant leaves (Buck and Andrews 1999; Gadanho and Sampaio 2005; Gadanho, Libkind and Sampaio 2006; Into *et al*. 2020). It is classified into the Sporidiobolaceae family, Sporidiobolales order, Microbotryomycetes class and Basidiomycota phylum. Some typical fungi in the Basidiomycota phylum include mushrooms, smuts and rusts. This classification makes *R. toruloides* unique, as many other yeasts for engineering studies such as *Saccharomyces cerevisiae*, *Lypomyces starkeyi* and *Y. lypolytica* are placed in the Ascomycota phylum. Because yeast species in the Pucciniomycotina of Basidiomycota phylum are polyphyletic, a recent phylogenetic classification proposes to rename *Rhodosporidium* as *Rhodotorula* (Wang *et al*. 2015). In this review, however, we used the name *Rhodosporidium toruloides* in order to better connecting with the majority of literature.

*Rhodosporidium toruloides* grows well at varying temperatures and pH (Gadanho, Libkind and Sampaio 2006). It uses diverse carbon sources as carbon and energy sources, for instance, monosaccharides including hexoses and pentoses; oligosaccharides such as sucrose, maltose, cellobiose, trehalose, raffinose and melezitose; alcohols such as ethanol, glycerol, mannitol and sorbitol; organic acids such as acetate, lactate, succinate, citrate and long-chain fatty acids as well as D-galacturonic acid (Yang *et al*. 2015; Xu and Liu 2017; Yaegashi *et al*. 2017; Jagtap and Rao 2018; Protzko *et al*. 2019). The robustness of *R. toruloides* in terms of resistance against biomass-derived inhibitors has been demonstrated (Hu *et al*. 2009; Zhao *et al*. 2012; Nogué *et al*. 2018). Thus, growing *R. toruloides* on biomass hydrolysates has been documented for production of lipids and carotenoids (Zhao *et al*. 2010; Matsakas *et al*. 2015; Singh *et al*. 2018; Dai *et al*. 2019; Osorio-González *et al*. 2019b; Bertacchi *et al*. 2020; Lopes, Bonturi and Miranda 2020). In terms of nitrogen sources, ammonium, nitrate, cadaverine, amino acids and small peptides are effective (Li, Zhao and Bai 2007; Wu *et al*. 2010; Li *et al*. 2020). To accumulate high amounts of lipids, oleaginous yeasts are usually cultivated under nitrogen-limited conditions (Evans and Ratledge 1984). Besides, limitation on other nutrients such as inorganic phosphate, sulfate and iron also facilitate lipid production by *R. toruloides* (Wu *et al*. 2010; Wu *et al*. 2011; Wang *et al*. 2018).

### Systems biology of *R. toruloides*

**Figure 1.**
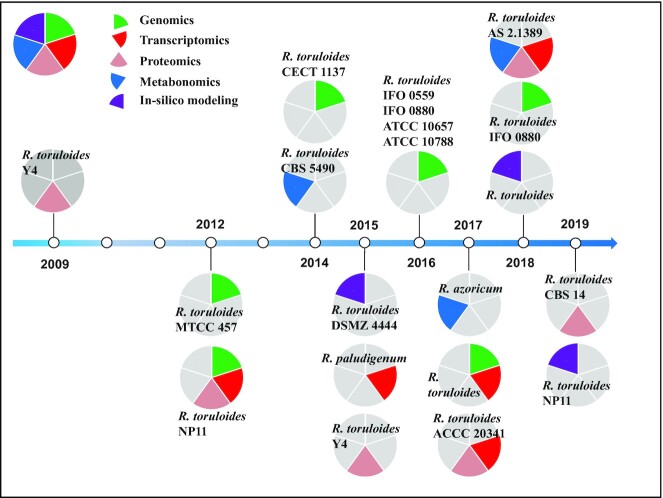
Milestones of systems biology studies in genus *Rhodosporidium*. The publication year, strain name and research approach are listed. Different colors are shown for genomics, proteomics, transcriptomics, metabolomics and in-silico modeling.

Genetic background and data in gene transcription, protein expression and basal metabolism are essential information for genetic modification and metabolic engineering. Significant progresses have been achieved in terms of understanding the genome, transcriptome, proteome and metabolome of *R. toruloides*, as summarized in Fig. 1 and Table S1 (Supporting Information).

#### Genome sequencing

Genome sequence is key to modern molecular biology of microorganisms. A genome sequencing study of *R. toruloides* NP11 by Zhao and his coworkers revealed a genome of 20.2 Mb in size with a GC content of 61.9% containing 8171 protein-coding genes (Zhu *et al*. 2012). Ever since, draft genomes of several *Rhodosporidium* species have been described (Kumar *et al*. 2012; Morin *et al*. 2014; Zhang *et al*. 2016c; Tran *et al*. 2019). More recently, two *Rhodosporidium* haploids with A1 and A2 mating type isolated from the same diploid strain were sequenced for comparison and sequencing data showed very close genome size (20.75 and 21.49 Mbp), GC content (62.0% and 61.8%) and protein-coding genes (7730 and 7800) (Hu and Ji 2016). With aids of functional genomics and comparative genomics, the chromosomal structure, protein-coding genes, predicted proteins and functional RNA of *Rhodosporidium* species can be elucidated.

#### Transcriptomics

The combination of genomics and transcriptomics facilitated the discovery of introns in the genome of *R. toruloides* NP11, and a novel fatty acid synthase system, which participates in metabolic pathways absent in non-oleaginous yeasts (Zhu *et al*. 2012). In other studies, transcriptomic studies were used to investigate gene expression profiles and global responses of *R. toruloides* to various stress conditions or utilization of a broad range of carbon sources (Zhu *et al*. 2012; Lu *et al*. 2015; Qi *et al*. 2017; Coradetti *et al*. 2018; Wang *et al*. 2018; Protzko *et al*. 2019). These efforts have identified some regulatory and stress-resistant genes.

#### Proteomics

Proteomic studies of *R. toruloides* have been adopted to compare differences in protein expression at diverse conditions. This technique is vital in providing a tool for the identification of proteins involved in lipid accumulation (Liu *et al*. 2009). In addition, it also help in the study of xylose metabolism (Tiukova *et al*. 2019a), and screening for dynamic changes of relevant proteins associated with lipid droplets (Zhu *et al*. 2015). Elsewhere, proteomic studies of the conventional yeast *S. cerevisiae* and two oleaginous yeast strains, *Cryptococcus albidus* and *R. toruloides*, provided an overall understanding of the excessive lipid storage in oleaginous yeasts (Shi *et al*. 2013). However, unlike isolated proteomic or transcriptomic analysis, integrated assay of proteomes and transcriptomes offers more evidence to identity key genes and pathways (Zhu *et al*. 2012; Qi *et al*. 2017).

#### Metabolomics

More recently, differences in titers and key metabolites under different culture conditions were investigated via metabolomics, which revealed the relevance between glycerol metabolism and carotenoid production in *R. toruloides* (Shen *et al*. 2017). Also, using metabolomics principles, the inhibition of glucose on glycerol metabolism, along with the importance of oxygen supply to the microbial lipid composition and yield was clarified (Lee *et al*. 2014). The analysis of metabolomes coupled with transcriptomes and proteomes revealed a comprehensive molecular mechanism of lipid accumulation during phosphorus depletion and predicted target genes for strain engineering (Wang *et al*. 2018).

#### Metabolic network reconstruction and modeling

**Figure 2.**
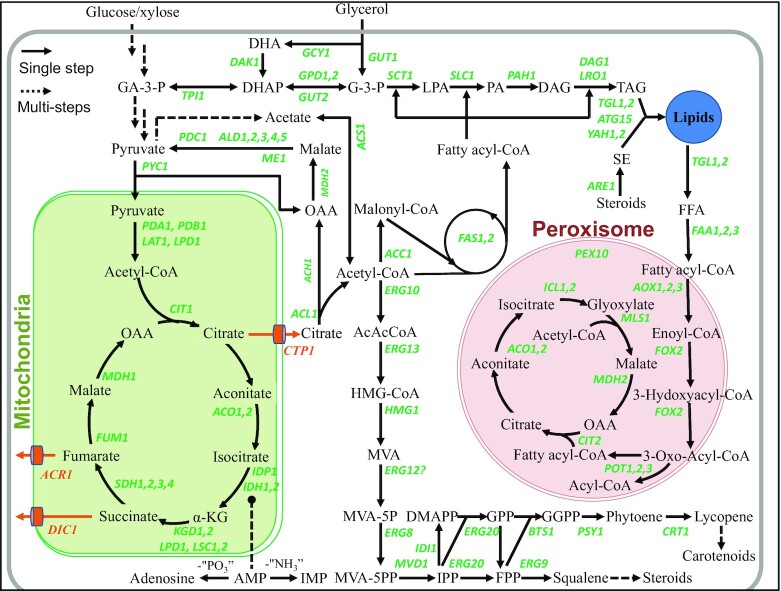
The primary metabolic pathways of *R. toruloides*. Key genes involved in glycolysis, pentose phosphate pathway, TCA cycle in mitochondria, biosynthesis and degradation of fatty acids, triacylglycerols and phospholipids, isoprenoid biosynthesis, glyoxylate cycle pathway, and the β-oxidation pathways in peroxisomes are highlighted in green. Abbreviations: GA-3-P, glyceraldehyde 3-phosphate; DHA, dihydroxyacetone; DHAP, dihydroxyacetone phosphate; G-3-P, glycerol-3-phosphate; LPA, lysophosphatidic acid; PA, phosphatidic acid; DAG, diacylglycerol; TAG, triacylglycerol; SE, steryl ester; FFA, free fatty acid; OAA, oxaloacetate; AHG, α-ketoglutarate; AcAcCoA, aceto-acetyl-CoA; HMG-CoA, hydroxymethylglutaryl-CoA; MVA, mevalonate; MVA5P, mevalonate-5-phosphate; MVA5PP, mevalonate-5-diphosphate; IPP, isopentenyl diphosphate; DMAPP, dimethylallyl diphosphate; GPP, geranyl diphosphate; FPP, farnesyl diphosphate; GGPP, geranylgeranyl diphosphate; AMP, adenosine monophosphate; IMP, inosine monophosphate.

Available omic studies and the framework of the molecular basis of the lipid metabolism, physiology and responses of *R. toruloides* to environmental stresses have provided datasets for metabolic network reconstruction and *in-silico* modeling (Zhu *et al*. 2012; Bommareddy *et al*. 2015; Castaneda *et al*. 2018; Coradetti *et al*. 2018; Dinh *et al*. 2019; Tiukova *et al*. 2019b). The primary metabolic pathways in cytoplasm, mitochondria and peroxisome are shown in Fig. 2.

According to the metabolism framework, acetyl-CoA is a key precursor of lipid synthesis, which mainly derives from the citric acid cleavage and pyruvate decarboxylation in the cytoplasm. Important genes involved in the conversion of acetyl-CoA into lipids, such as *ACC*1 (coding acetyl-CoA carboxylase), *SCD*1 (coding stearic CoA desaturase) and *DGAT*1 (coding diacylglycerol acyltransferase) are well characterized and presumed to catalyze the first, intermediate and final step, respectively, in triacylglycerol biosynthesis (Yang *et al*. 2012; Chen *et al*. 2014; Cui *et al*. 2016; Liu *et al*. 2016b). NADPH, a reducing power used for fatty acid acyl carrier protein synthesis, springs from the oxidative pentose phosphate pathway, and the POM cycle (pyruvate/oxaloacetate/malate cycle or transhydrogenase cycle) via cytosolic NADP^+^-dependent malic enzyme (Zhu *et al*. 2012; Coradetti *et al*. 2018). Acetyl-CoA can also be used to synthesize dimethylallyldiphosphate and its isomer 3-isopentenyl diphosphate—the common precursors of terpenoids, for instance, lycopene and β-carotene. Free fatty acids are also degraded in peroxisome to generate acetyl-CoA which could be converted into succinate by the glyoxylate cycle (Bommareddy *et al*. 2015; Castaneda *et al*. 2018).

In addition to the basic metabolic network, the effects of nitrogen and phosphorus depletion on lipid accumulation and relevant metabolism have been explored by system biology study (Zhu *et al*. 2012; Wang *et al*. 2018). Nitrogen or phosphorus limitations are linked to intracellular adenosine monophosphate (AMP) which converts to inosine monophosphate (IMP) through deamination or to adenosine (Ade) through dephosphorylation. These processes impair the activity of mitochondrial isocitrate dehydrogenase (Idh) owing to its high dependence on AMP. Low Idh activity is also implicated to influence the accumulation of mitochondrial citrate and its export to the cytoplasm, with further cleavage of citrate into OAA and acetyl-CoA for enhanced lipid production. In principle, nitrogen starvation promotes the up-regulation of genes involved in fatty acid synthesis and decomposition pathways. The pentose phosphate pathway and the transhydrogenase pathway increase NADPH supply and basically enhance the synthesis of triacylglycerols. Nitrogen starvation makes the cells prone to autophagy (Zhu *et al*. 2012) whereas the autophagy and RNA degradation pathways get activated in phosphorus exhaustion. Simultaneously, the expression of phosphatidate phosphatase (Pah1) and diacylglycerol acetyltransferase (Dag1) are significantly up-regulated while the β-oxidation pathway gets inhibited. These regulatory proteins and pathways contribute to the synthesis of triacylglycerol (Wang *et al*. 2018).

Recent studies on the genome, transcriptome, proteome and metabolome of *R. toruloides* are summarized in Table S1 (Supporting Information). It shows a trend of multi-omic analysis, because the results obtained from different omic layers can be reciprocally verified and provide more reliable conclusions. However, very few integrated studies of various omics and models are reported (Bommareddy *et al*. 2015; Castaneda *et al*. 2018). A curated version of a small-scale metabolic model for de novo lipid production by *R. toruloides* showed that the central nitrogen metabolism essential to predict the lipid metabolism at different culture conditions was unblocked (Castaneda *et al*. 2018). Moreover, the process of mass and charge balancing incorporated additional constraints on cell mass production. With the accumulation of omic data, as well as advances in technologies such as big data analysis and artificial intelligence, multi-omics research will pave the way and provide clues for genetic technology development, metabolic engineering, evolutionary engineering and process engineering.

### Genetic tools development

Omics study promoted the discovery of biological elements such as replicons, promoters, terminators and selection markers for *R. toruloides*. However, there was no endogenous plasmid or autonomously replicating sequence and centromere sequence functional as a replication element being verified in *R. toruloides*.

#### Transformation methods

Genetic transformation was first reported with random integration of the phenylalanine ammonia lyase (*PAL*) gene into the chromosome of *R. toruloides* MS7013 through polyethylene glycol (PEG)-mediated protoplast transformation with a transformation efficiency of about 1000 transformants/μg DNA (Tully and Gilbert 1985). Because protoplast transformation efficiency is highly dependent on protoplast preparation and operation, subsequent studies sought to use *Agrobacterium tumefaciens*-mediated transformation (ATMT) (Liu *et al*. 2013). ATMT transformation is widely applicable, and the transformation efficiency varies from 70 to 1000 transformants per plate depending on the species and selection markers (Koh *et al*. 2014; Lin *et al*. 2014). Even though ATMT is easy to operate, its process is time-consuming as a single round preparation takes almost 2 weeks (Sun *et al*. 2017; Hooykaas *et al*. 2018). Moreover, as the intrinsic feature of ATMT transformation is random integration of the genome, it is essential to screen massive clones to identify those with superior phenotype and to diminish the risk of unexpected interference with endogenous genes (Wang *et al*. 2017).

Lithium acetate/PEG-mediated chemical transformation methods have also been evaluated for genetic transformation of *R. toruloides* DMKU3-TK16. In this technique, the transformation protocol is easy to master, even as it is currently hampered by the low efficiency of about 25 transformants/μg DNA (Tsai *et al*. 2017). More recently, electroporation has been adopted to transform *R. toruloides* (Liu *et al*. 2017), with a varied transformation efficiency of 40–1000 CFU/μg DNA depending on the strains.

The outcomes and features of different transformation methods applied in *R. toruloides* were compared in Table S2 (Supporting Information). It is clear that various methods have been attempted for random integration and site-specific deletion, with wide-ranging efficiency. Currently, ATMT is still the most reliable method, despite problems of T-DNA integration into the genome with a high probability of random insertion via non-homologous end joining (Koh *et al*. 2014; Sun *et al*. 2017; Wang *et al*. 2017). This method leads to a very low proportion of mutants generated from homologous recombination, thus necessitating the quest for the designing of some new methods to effectively filter out false-positive mutants. Previous research suggested that once a counter selection marker or a lethal gene such as herpes simplex virus thymidine kinase gene gets randomly integrated into chromosome by ATMT, the mutants get hardly grown in counter-screening medium (Khang *et al*. 2005). This phenomenon can be applied to filter ectopic transformants, while preserving the target mutants. Plenty of studies have illustrated strategies of transferring Ti plasmids harboring counter selection marker cassette locating outside homologous arms. This technique has proved helpful in the screening of positive mutants in other fungi including *Magnaporthe grisea*, *Fusarium oxysporum* and *Verticillium dahliae* (Khang *et al*. 2005; Tian *et al*. 2011; Jiang *et al*. 2013), which could be used to improve ATMT in *Rhodosporidium* species.

Generally, low transformation efficiency is one of the bottlenecks for all gene transfer methods. The highest transformation efficiency of *R. toruloides* is usually at the level of 10^3^ transformants/μg DNA (or 10^5^ cells), which makes the success of genetic manipulation much dependent on tools with higher transformation efficiency such as transposon-mediated mutations (Kumar 2016). It is important to note the difficulty to obtain a favorable mutant when the positive frequency is below 10^−3^. As a result, a lot of manpower and resources are committed to increasing transformants number, as the background ectopic mutants need to be filtered out by means of counter-screening and other methods. Besides (non-)homologous recombination of *R. toruloides*, there are also a few reports on the site-specific integration and the in-frame deletion of target genes free of scar (Koh *et al*. 2014; Sun *et al*. 2017; Sun *et al*. 2018).

#### Genetic parts

Like in other eukaryotes, a gene expression module in *R. toruloides* also follows a pattern of ‘promoter-functional gene-terminator’, in which the ‘functional gene’ includes selection markers, reporter genes and those coding for dedicated proteins. A number of toolsets such as promoters, terminators, selection markers and reporter genes have been verified functional in *R. toruloides*, as shown in Table S3 (Supporting Information). Endogenous constitutive and inducible promoters could be predicted from RNA sequencing data, as seen by a recent example of identification of 12 monodirectional and 8 bidirectional native promoters for *R*. *toruloides* (Nora *et al*. 2019).

Usually, the strength of promoters is characterized by the expression levels of reporter genes or resistance markers. For instance, the strength order of the constitutive promoters of P*_PGI_*, P*_PGK_*, P*_FBA_*, P*_TPI_* and P*_GPD_* in *R. toruloides* Y4 was determined through comparing the expression level of hygromycin resistance gene driven by these promoters (Wang *et al*. 2016b). In another study, the P*_GPD1_* promoter of *R. toruloides* ATCC 10657 was identified and luciferase was used as a reporter gene to measure the strength of the 6 promoters regulating important genes involved in the lipid synthesis by *R. toruloides* (Liu *et al*. 2016a). Among them, the strength of the P*_LDP1_* promoter is up to10-fold higher than that of the P*_GPD1_* promoter, which has generally been considered as one of the strongest promoters in yeast. In addition, a P*_DAO1_* promoter system that can be induced by D-amino acids was developed (Liu *et al*. 2015). Depending on the concentration of the inducer (such as D-alanine), the strength of the P*_DAO1_* promoter can vary up to 10-fold, implying very good potential in gene expression regulation. Furthermore, inducible promoters P*_PHO89_*, P*_ADH2_* and P*_GAL1_*were identified, characterized and tightly regulated by phosphate and glucose concentration (Ma 2015). More recently, new inducible promoters such as P*_NAR1_*, P*_ICL1_*, P*_CTR3_* and P*_MET16_* have been isolated which further enriched the repertoire of tunable genetic components (Johns, Love and Aves 2016). The promoter of RNA polymerase III was also identified that was used to express small RNA such as sgRNA (Jiao *et al*. 2019; Schultz, Cao and Zhao 2019).

In addition to the promoter from the genome, the virus 2A sequence is also effective to mediate the co-expression of multiple genes in *R. toruloides* (Jiao *et al*. 2018). Two different 2A sequences, porcine teschovirus-1 2A (P2A) and foot-and-mouth disease virus 2A (F2A) were evaluated as genetic elements to drive co-expression of the gene fused downstream the 2A sequence.

Terminators are specific DNA sequences within a few hundred base pairs in length and are needed to terminate gene transcription. As usual, terminators are located downstream of the gene stop codon, with feature sequences of (T-rich).TA (T) GT.(AT-rich)..TTT and TTTTTATA. Several endogenous terminators of genus *Rhodosporidium*, such as T*_hsp_*, T*_gpd_*, etc., have been applied for gene expression termination (Jiao *et al*. 2018). Some heterogeneous terminators like T_35s_ from *Cauliflower mosaic virus* and T*_nos_* from *A. tumefaciens* have also been verified or reported functional in *R. toruloides* (Diaz *et al*. 2018).

Like promoters and terminators, selection markers and reporter genes also play crucial roles in genetic engineering. The available auxotrophic markers in genus *Rhodosporidium* are *URA3* and *LEU2*, while that of antibiotics resistance include *HYG*, *BLE*, *NAT* and *G418*. Multiple rounds of integration experiments indicated that antibiotics resistance markers *HYG*, *BLE* and *NAT* could work independently in *R. toruloides* (Yang *et al*. 2008; Lin *et al*. 2014). Reporter genes such as luciferase- and green fluorescent protein (GFP)-coding genes have been applied in promoter characterization and strain screening (Liu *et al*. 2013; Liu *et al*. 2015; Johns, Love and Aves 2016; Diaz *et al*. 2018).

As summarized in Table S3 (Supporting Information), genetic parts are now largely in *R. toruloides*. Yet, some other elements such as nuclear localization sequence (NLS) and the application of other common reporter genes such as YFP and auxotrophic markers such as HIS remain to be demonstrated.

#### Targeted genome editing

RNA interference (RNAi) is a biological process in which RNA molecules inhibit gene expression or translation, by neutralizing targeted mRNA molecules. RNAi is one of the primary genetic tools that are independent of homologous recombination. It has been demonstrated that RNAi machinery is functional for gene-downregulation in *R. toruloides* (Liu *et al*. 2019). Inactivation of specific endogenous genes responsible for easy-screening phenotypes and genes overexpression by random integration has also been demonstrated (Koh *et al*. 2014; Sun *et al*. 2017). However, in-frame deletion or targeted integration process remains challenging.

One of the primary challenges of precise genetic modification is the low homologous recombination efficiency. When the knockout cassette of phytoene dehydrogenase gene *CRT1* was introduced in *R. toruloides* NP11 through the ATMT method, only about 2% transformants were found with homologous recombination (Sun *et al*. 2017). Similarly, knocking out the gene *CAR2* (encoding carotene cyclase) in the wild-type strain *R. toruloides* ATCC 10657 using the ATMT mediated homologous recombination gave a positive rate of 10.5% (Koh *et al*. 2014). In the Ku70-deficient strain *R. toruloides* ATCC 10657 *△*ku70, the targeted deletion frequency increased to 75.3% but the total number of transformants decreased to 14% of that of the wild-type strain. This suggested that the knockout of Ku70 reduces the ability of T-DNA to randomly insert into the chromosome, thereby excluding a large number of ectopic mutants. Thus, it seemed that Ku70 knockout decreased the screening workload with limited contribution to homologous recombination. Native homologous recombination factors or orthologous genetic parts such as *RAD52* from *S. cerevisiae* are required to further improve homologous recombination in *R. toruloides* (van Attikum and Hooykaas 2003; Rolloos *et al*. 2014; Hooykaas *et al*. 2018).

As low homologous recombination efficiency and low transformation efficiency collectively limit the development of genetic tools based on homologous recombination, the application of other approaches especially the CRISPR-Cas system in *Rhodosporidium* becomes important. Very recently, three research groups independently developed the CRISPR-Cas9 system applicable in *R. toruloides* (Jiao *et al*. 2019; Otoupal *et al*. 2019; Schultz, Cao and Zhao 2019). These methods adopted different sgRNA transcription strategies to assist in Cas9 editing, hence achieving single or multi-genes editing via homologous recombination or NHEJ. However, the current technology is shrouded with several putative risks. First, both Cas9 and sgRNA cassettes are randomly integrated into the genome via ATMT or lithium chloride transformation, which may result in the inactivation of genes locating at the integration site. It could also be linked to the integrative and constitutive expression of sgRNA which result in off-target or toxicity of Cas9.

Since no replicable plasmid is available in *R. toruloides*, a gene of interest must be integrated into the chromosome for its function analysis. A classical Cre/loxP site-specific recombination system with the introduction of the Flp/FRT recombinase system was also applied to realize multiple rounds of random integration of heterogeneous genes (Sun 2017). Elsewhere, other recombinases like I-SceI have been adapted for the recovery of the G418 resistance marker in *R. toruloides* CECT 13085 (Fillet *et al*. 2016). In general, the application of recombinases implied that it is possible to achieve in-frame deletion and targeted insertion in the genome.

Currently, genetic parts, transformation methods and genetic tools remain to be enriched for complicated strain engineering of *R. toruloides*. Fortunately, many of those functional in other yeasts have been introduced successfully into genus *Rhodosporidium* (Lin *et al*. 2014; Johns, Love and Aves 2016; Wang *et al*. 2016b). Ultimately, mining, characterization and standardization of genetic toolsets will no doubt boost the emergence of novel gene-editing technologies which improve efficiency of strain engineering study.

### Strain engineering

**Figure 3.**
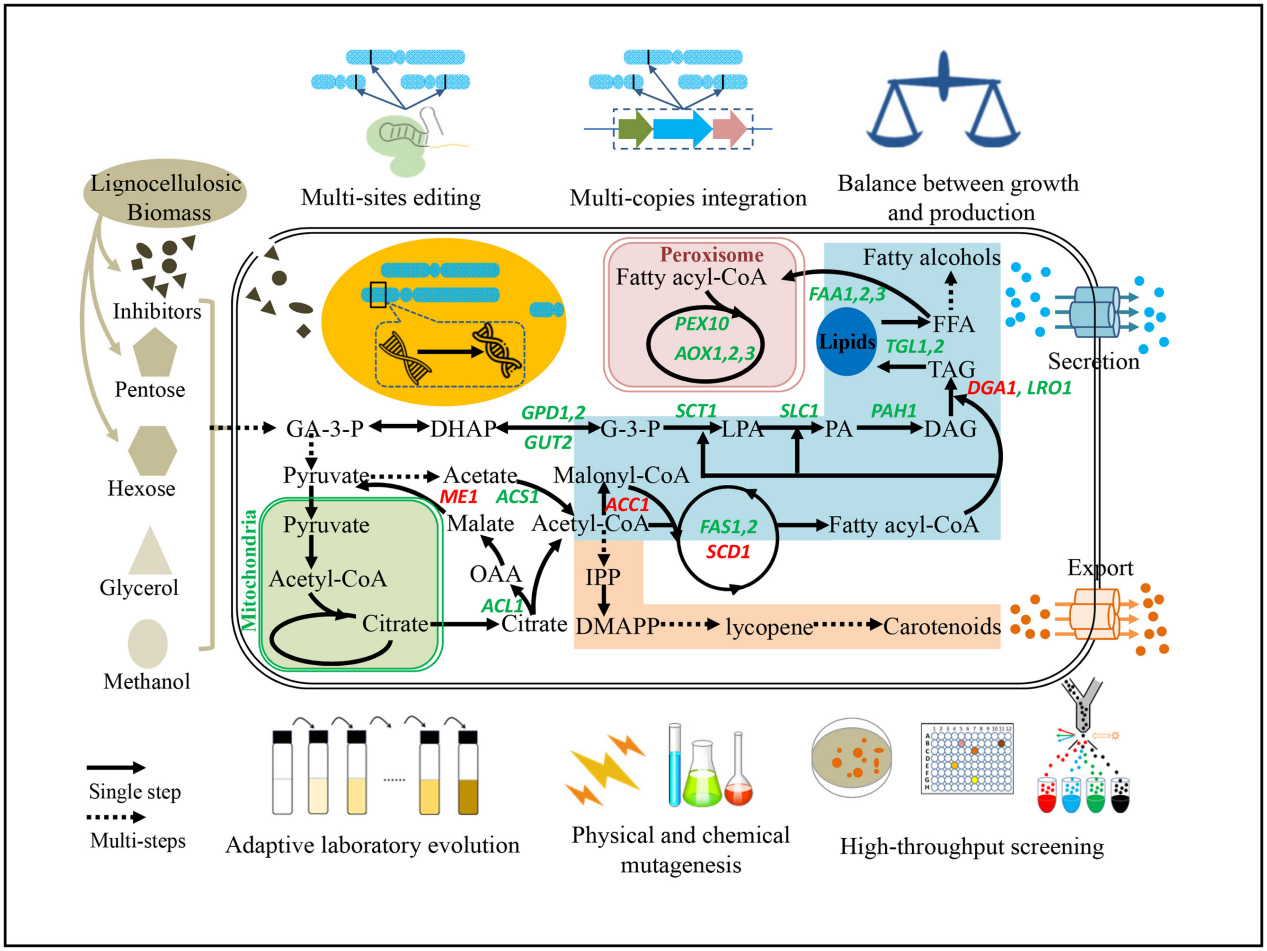
Strains engineering of genus *Rhodosporidium* as microbial platform. With the aid of rational and semi-rational approaches, including metabolic engineering, adaptive laboratory evolution, physical & chemical mutagenesis, high-throughput screening, *Rhodosporidium* is expected to be engineered to utilize lignocellulosic biomass, glycerol and methanol to produce and export fatty acid derivatives (highlighted in blue), terpenoids (highlighted in light orange). Key genes involved in fatty acid derivatives are highlighted in green and red (identified and characterized well in literature). Abbreviations: GA-3-P, glyceraldehyde 3-phosphate; DHAP, dihydroxyacetone phosphate; G-3-P, glycerol-3-phosphate; LPA, lysophosphatidic acid; PA, phosphatidic acid; DAG, diacylglycerol; TAG, triacylglycerol; FFA, free fatty acid; OAA, oxaloacetate; IPP, isopentenyl diphosphate; DMAPP, dimethylallyl diphosphate.

Increasingly enriched knowledge at systems level has been facilitating metabolic engineering of *R. toruloides* for diverse products including fatty acid derivatives, carotenoids, terpenoids and blue pigments. Similarly, efforts are devoted to expanding the substrate spectrum and stress resistance space (Fig. 3). A brief list of these examples is also shown in Table S4 (Supporting Information). In fact, the potential of *R. toruloides* as a microbial platform in biotechnology has also been reviewed recently (Xu and Liu 2017; Park, Nicaud and Ledesma-Amaro 2018; Saini *et al*. 2020).

#### Engineering for fatty acid derivatives

Manipulation of important genes related to lipid biosynthesis has been successful in several aspects. Engineered *R. toruloides* strains were found with significantly increased lipid contents and yields under phosphorus-limited conditions upon expression of the key enzyme malic enzyme (Me) for improved NADPH supply (Wang *et al*. 2018). This is in-line with another work in which over-expression of Me in *R. toruloides* IFO 0880 boosted lipid production by 24% under nitrogen-limited conditions (Zhang *et al*. 2016b). In *R. toruloides* IFO 0880, random integration of genes encoding Acc1 and Dag1 led to increasing lipid content by 49% and lipid titer by 74% (Zhang *et al*. 2016c). Random integration of endogenous genes *DGAT1* and *SCD1* into *R. toruloides* CECT 13085 revealed an enhanced lipid yield and titer by 13% and 13%, respectively, while engineered strains produced about 39 g/L of lipid from lignocellulosic biomass hydrolysates (Diaz *et al*. 2018). As acetyl-CoA is a sole precursor to fatty acid biosynthesis, many studies have known to enhance its supply for improved production of fatty acid derivatives by in *S. cerevisiae* or *Y. lipolytica* (Xu *et al*. 2016; Zhou *et al*. 2016b; Qiao *et al*. 2017; Yu *et al*. 2018). In *R. toruloides* NP11, the introduction of phosphotransacetylase-coding gene from *Bacillus subtilis* to establish the conversion of phosphoacetate into acetyl-CoA led to increasing cell mass, lipid yield and titer by 26%, 11% and 54%, respectively (Yang *et al*. 2018).

Another scenario is to engineer advanced strains for the production of high-valued lipids. Previous studies demonstrated the composition of neutral lipids of *R. toruloides* as follows: palmitic acid 23%–30%, oleic acid (OA) 30%–37%, stearic acid 32%–37%, and linoleic acid 2%–4% (Papanikolaou and Aggelis 2011). To improve OA contents, *R. toruloides* strains were engineered by integrating an expression cassette for the Δ9-fatty acid desaturase from *S. cerevisiae* or the endogenous Δ9-fatty acid desaturase, leading to more than 5-fold higher OA contents, i.e. over 70% of the fatty acid composition of the total lipids (Wang *et al*. 2016a; Tsai *et al*. 2019). For linoleic acid production, an expression cassette for Δ12-fatty acid desaturase was integrated into the diploid strain *R. toruloides* AS 2.1389, thus achieving a titer of 1.3 g/L (Wang *et al*. 2016a). Random insertion of genes encoding Δ12 desaturase and ω-3 desaturase in combination with aldehyde dehydrogenase in-frame deletion afforded *R. toruloides* strains capable of producing lipids with α-linolenic acid content of ∼49% of total fatty acids (Fillet *et al*. 2016). More recently, genes encoding 3-ketoacyl-CoA synthase from different plants were genetically integrated into *R. toruloides* CECT 13085 and the recombinant strains produced very long-chain fatty acids, erucic acid and nervonic acid for the first time (Fillet *et al*. 2017). It was found that the titers of erucic acid and nervonic acid were stimulated upon increasing the copy numbers of the 3-ketoacyl-CoA synthase gene.

By overexpression of fatty acyl-CoA reductase from *Marinobacter aquaeolei* VT8 in *R. toruloides* CECT 13085, the engineered strain produced up to 8.0 g/L of long-chain fatty alcohols with 16–18 carbon atoms upon cultivation on sucrose for 75 h in a 7-L bioreactor (Fillet *et al*. 2015). By using nonionic surfactants during the culture process, fatty alcohol production was further enhanced by engineered strains (Liu *et al*. 2020).

#### Engineering for carotenoids and terpenoids

One key feature of *R. toruloides* is to accumulate both triacylglycerides and carotenoids (Buzzini *et al*. 2007; Singh *et al*. 2016; Singh *et al*. 2018; Bertacchi *et al*. 2020; Tran *et al*. 2020). Some efforts have been done by regulation of its endogenous enzymes to enhance carotenoids biosynthesis (Sun *et al*. 2017; Liu *et al*. 2018; Jiao *et al*. 2019). Attempts have also been tried to use *Rhodosporidium* as a production host for terpene-based biofuels by evaluation of 16 terpene synthases (TS) from plants, bacteria and fungi. Eight of these TS were found functional in *R. toruloides* and a total of nine different monoterpenes were obtained. The engineered strain produced either a single terpene compound or mixtures of others such as 1,8-cineole, sabinene, ocimene, pinene, limonene, and careen (Zhuang *et al*. 2019). Similarly, *R. toruloides* has been engineered for the production of other terpenoids including bisabolene, amorphadiene and *ent*-kaurene. When cultivated on the alkaline corn stover hydrolysate, bisabolene was produced by the engineered strain at a titer of over 680 mg/L; while on a glucose-only medium, it was at 521 mg/L (Yaegashi *et al*. 2017). Ionic liquids-rich hydrolysates were also feasible substrates for the *R. toruloides* strain to produce bisabolene, demonstrating a good compatibility with biomass pretreatment method (Sundstrom *et al*. 2018). Furthermore, bisabolene titers were improved to 2.2 g/L in a 20-L bioreactor using separation-free biomass hydrolysates as substrate (Pimienta *et al*. 2019). The diterpene *ent*-kaurene was produced in a 2-L bioreactor at a titer of 1.4 g/L from corn stover hydrolysates by engineered *R. toruloides* strain that expressed genes encoding the kaurene synthase from *Gibberella fujikuroi* and a mutated farnesyl diphosphate synthase from *Gallus gallus* for enhanced geranylgeranyl diphosphate supply (Geiselman *et al*. 2020).

#### Engineering for other metabolites

Indigoidine is a natural blue pigment produced by several bacteria including *Streptomyces lavendulae* and its biosynthetic gene cluster has been well established. This 3′,3′-bipyridyl pigment is formed through condensation of L-glutamine catalyzed by a non-ribosomal peptide synthetase (NRPS). Interestingly, by expressing the *BpsA* gene cluster from *S. lavendulae* in *R. toruloides* IFO 0880, the native metabolite pools of glutamine were converted into indigoidine. The engineered strain produced indigoidine at a titer of 2.9 g/L using a sorghum hydrolysate as carbon sources in a batch process and the highest titer of 86 g/L on glucose in a high-gravity fed-batch process within a 2-L bioreactor (Wehrs *et al*. 2019). This study further demonstrated the potential of *R. toruloides* for biotechnological applications.

It should also be noted that *R. toruloides* wild-type strains have been found as workhorses to produce sugar polyols (Jagtap and Rao 2018; Jagtap *et al*. 2019) and important enzymes.

#### Engineering with traditional approaches

It should be mentioned that traditional approaches such as physical or chemical mutagenesis and adaptive laboratory evolution have also been used for strain engineering. For example, UV mutagenesis, chemical mutagenesis along with atmospheric and room temperature plasma methods are used to mutate *R. toruloides* for enhanced lipid or carotenoid production (Qi *et al*. 2014; Zhang *et al*. 2016a; Guo *et al*. 2019; Tran *et al*. 2020). These approaches have been explored to identify strains with superior physiological features and then putative clues may be delineated via systems biology analysis for reverse metabolic engineering studies (Yamada, Kashihara and Ogino 2017).

### Future directions

Apparently, *R. toruloides* has attracted major interests in academia and industry due to its unique physiological traits and vast imbedded potentials (Xu and Liu 2017; Park, Nicaud and Ledesma-Amaro 2018; Osorio-González *et al*. 2019a; Saini *et al*. 2020). Our knowledge has been substantially enriched in terms of the molecular basis of the genome, metabolism and physiology. Reliable toolsets are now accessible for strain engineering and systems biology studies. To facilitate more fruitful researches with this red yeast, several directions are worthwhile mentioning.

First of all, more efficient genetic tools should be developed. Albeit many genetic parts are available and genome editing methods have been documented very recently, the overall efficiency remains considerably lower to engineer *R. toruloides* than several other yeasts including *S. cerevisiae* and *Y. lipolytica*. This is largely due to the low efficiency of homologous recombination and transformation, and the absence of replicable plasmids for *R. toruloides*. In this regard, strains with much improved homologous recombination should be designed and evaluated. Of course, it is always valuable to characterize more genetic parts and devise more effective transformation protocols. Of special interests is to further customize the CRISPR-Cas9 system recently developed for targeted genome editing of *R. toruloides*.

Second, fundamentals on the systems biology of lipid production should be emphasized. Although lipid biology related to the health issues has been intensively studied at the systems biology level, efforts are limited to address those issues of truly biotechnology-relevant. As an excellent lipid producer, *R. toruloides* is a wonderful eukaryotic organism to understand the sophisticated regulatory circuits and networks related to lipid metabolism such as lipid droplet dynamics, lipid transportation and the relationships among lipid accumulation, autophagy and senescence. The analysis of existing and to-be-generated omics data with advanced bioinformatics tools will be fruitful in this subject. Another topic of great engineering interests is the mechanism regulating fatty acid biosynthesis and isoprenoid biosynthesis. Noting that acetyl-CoA is the central precursor to fatty acids and isoprenoids, both of them being produced by *R. toruloides* natively, the potential to direct acetyl-CoA for production of isoprenoids in high yields remains to be demonstrated.

Third, more efforts should be devoted to devising *R. toruloides* strains for high-value products. So far, limited success has been known for the synthesis of unsaturated fatty acids, fatty alcohols, carotenoids and blue pigments. Of special interests is to engineer the fatty acid biosynthesis machinery for designer chemicals as demonstrated recently in *S. cerevisiae* (Zhu *et al*. 2017). Although the main stream is to engineer *R. toruloides* for functional lipids, the potential has been documented to use this yeast for the production of non-ribosomal peptides and sugar alcohols. While the integration of key functional genes into the genome has been readily accessible to deliver new products, more studies should also consider pathway bypass, cofactor balance, regulatory genes and subcellular organelle engineering as seen in other yeasts (Zhou *et al*. 2016a; Qiao *et al*. 2017).

Lastly, it is almost always overlooked but in fact essential to engineer the physiology beneficial to the techno-economics of microbial lipid production. Some very important traits including but not limited to secretory lipid production, stress resistance to high temperatures, extreme pH and high osmotic pressure and expanded substrate scope to organic components found in typical biomass hydrolysates. For example, a membrane transporter was used to export lipid products from *R. toruloides* cells into the medium in a bi-phasic culture for improved product separation and yields (Lee *et al*. 2016). *R. toruloides* strains with improvements in these features are much more attractive for applications to use the real world low-cost feedstocks. Yet, this is very challenging as the desired strains are expected to result from systematic and global rewiring of cellular metabolism.

Together, *R. toruloides* is expected to subject rational genetic engineering and semi-rational approaches integrated with high-throughput screening for advanced strains in diverse biotechnology applications of great academic and economic value. Concurrently, our understanding on the fundamentals of physiology and biology of this yeast will be enriched.

## Supplementary Material

foaa038_Supplemental_File
