# Supplementary material for: Rhodosporidium toruloides - A potential red yeast chassis for lipids and beyond
Source: FEMS Yeast Res. 2020 Jul 2;20(5):foaa038. doi: 10.1093/femsyr/foaa038 (PMC7334043; doi:10.1093/femsyr/foaa038)
Supplement: foaa038_Supplemental_File [file foaa038_supplemental_file.docx]

Table S1. Summary of completed and initiated omics projects in *Rhodosporidium* species. Data is adapted from the National Center for Biotechnology Information (NCBI) or European Nucleotide Archive (ENA).

| Omics | Strains | Description | | | | | | References |
| --- | --- | --- | --- | --- | --- | --- | --- | --- |
|  |  | Accession number | Size | Protein-encoding gene | GC% | Update Date | Create Date |  |
| Genomics | *R. toruloides* MTCC 457 | AJMJ00000000 | 20.09 Mb | 5993 | 62.0% | 2014-08-11 | 2012-04-13 | Kumar et al. 2012 |
|  | *R. toruloides* ATCC 10657 | LNQQ00000000 | 20.75 Mb | 7730 | 62.01% | 2016-04-07 | 2016-01-27 | Hu and Ji 2016 |
|  | *R. toruloides* ATCC 10788 | LNKU00000000 | 21.49 Mb | 7800 | 61.81% | 2016-04-07 | 2016-01-27 | Hu and Ji 2016 |
|  | *R. toruloides* CECT 1137 | LK052936 to LK052997 | 20.44 Mb | 8206 | 61.9% | 2014-06-05 | 2014-06-05 | Morin et al. 2014 |
|  | *R. toruloides* IFO 0880 | LCTV00000000 | 20.68 Mb | 8490 | 61.9% | 2018-03-14 | 2018-03-14 | Zhang et al. 2016 |
|  | *R. toruloides* IFO 0559 | LCTU00000000 | 20.28 Mb | 8100 | 61.9% | 2015-05-06 | 2015-05-06 | Zhang et al. 2016 |
|  | *R. toruloides* NP11 | ALAU00000000 | 20.18 Mb | 8171 | 61.9% | 2014-08-04 | 2012-10-30 | Zhu et al. 2012 |
|  | *R. toruloides* ATCC 204091 | AEVR00000000 | 20.48 Mb | 6631 | 61.9% | 2014-08-11 | 2013-12-03 | Paul et al. 2014 |
|  | *R. graminis* WP1 | JTAO00000000 | 20.78 Mb | 7278 | 67.8% | 2015-10-19 | 2015-10-14 | Firrincieli et al. 2015 |
|  | *R. mucilaginosa* C2.5t1 | JWTJ00000000 | 20.0 Mb | 6413 | 60.5% | 2015-04-15 | 2015-02-24 | Deligios et al. 2015 |
|  | *Rhodotorula sp.* JG-1b | LQXB00000000 | 19.4Mb | 6681 | 60.6% | 2016-04-07 | 2016-01-26 | Goordial et al. 2016 |
|  | *R.taiwanensis* MD1169 | PJQD00000000 | 19.6 Mb | 7014 | 61.6% | - | 2018-02-07 | Tkavc et al. 2018 |
|  | *R. toruloides* VN1 | SJTE00000000 | 20.01 Mb | 8021 | 61.8% | - | 2019-03-03 | Tran et al. 2019 |
| Transcriptomics | *R. toruloides* NP11 | Transcriptomic analysis of *R. toruloides* under nitrogen starvation | | | | | | Zhu et al 2012 |
|  | *R. toruloides* ACCC 20341 | Transcriptomic analysis of *R. toruloides* mutants that tolerate lignocellulosic hydrolysates | | | | | | Qi et al. 2017 |
|  | *R. toruloides* AS 2.1389 | Differential transcriptomic analysis in response to phosphate-limitation | | | | | | Wang et al. 2018 |
| Proteomics | *R. toruloides* NP11 | Protein expression analysis under nitrogen starvation | | | | | | Zhu et al 2012 |
|  | *R. toruloides* CBS 14 | Protein expression analysis of *R. toruloides* during conversion of xylose to lipids | | | | | | Tiukova et al. 2019a |
|  | *R. toruloides* ACCC 20341 | Proteomic analysis of *R. toruloides* mutants that tolerate lignocellulosic hydrolysates | | | | | | Qi et al. 2017 |
|  | *R. toruloides* Y4 | Differential proteomic analysis of lipid accumulation | | | | | | Liu et al. 2009 |
|  | *R. toruloides* AS 2.1389 | Differential proteomic analysis in response to phosphate-limitation | | | | | | Wang et al. 2018 |
|  | *R. toruloides* ACCC 10788 | Differential proteomic analysis of lipid accumulation | | | | | | Shi et al. 2013 |
|  | *R. toruloides* AS 2.1389 | Proteomic analysis of lipid droplets and their dynamic changes during lipid accumulation | | | | | | Zhu et al. 2015 |
| Metabonomics | *R. azoricum* | Metabolomic analysis of lipid accumulation in the absence of nitrogen and oxygen | | | | | | Capusoni et al. 2017 |
|  | *R. toruloides* CBS 5490 | Metabolomic analysis emphasizing glycerol utilization, TCA cycle and carotenoid synthesis | | | | | | Lee et al. 2014 |
|  | *R. toruloides* AS 2.1389 | Monitoring changes in marker metabolites and analyzing cellular metabolic response to phosphorus deficiency | | | | | | Wang et al. 2018 |
| In-silico modeling | *R. toruloides* DSMZ 4444 | The model includes 69, 71, 71 and 72 reactions respectively with glucose, glycerol, xylose and arabinose as sole carbon source | | | | | | Bommareddy et al. 2015 |
|  | *R. toruloides* | The model includes 93 metabolites and 104 reactions unblocking the central nitrogen metabolism and completing charge and mass balances | | | | | | Castaneda et al. 2018 |
|  | *R. toruloides* NP11 | The model includes 852 genes, 2,731 reactions, and 2,277 metabolites | | | | | | Tiukova et al. 2019b |

Table S2. Summary of transformation method development in genus *Rhodosporidium*.

| Transformation methods | Strains | Description | Characteristics | References |
| --- | --- | --- | --- | --- |
| PEG-mediated protoplast transformation | *R. toruloides* MS7013 (PAL^-^) | 1. Random integration of PAL genes  2. Transformation efficiency is 1000 transformants /μg DNA | 1. Transformation operation is laborious  2. Transformation efficiency is highly dependent on protoplast | Gilbert, 1985 |
| ATMT (Random insertion) | *R. toruloides* ATCC 10657 | 1. Random integration of GFP reporting system and resistance marker cassette  2. Transformation efficiency is 1000 transformants / plate | 1. Transformation operation is cumbersome but easy  2. The transformation efficiency is affected by T-DNA plasmid, *Agrobacterium* and *Rhodosporidium* species, and resistance marker.  3. Gene is randomly inserted into the chromosome  4. ATMT can be performed successively | Liu et al. 2013 |
|  | *R. toruloides* NP11  *R. toruloides* Y4 | 1. Randomly integrate HYG, NAT, and BLE resistance marker cassette successively  2. Transformation efficiency varying from 70-1000 transformants/10^5^ cells |  | Lin et al. 2014 |
| ATMT (targeted deletion) | *R. toruloides* NP11 | Directly target CRT1 gene with the HYG resistance cassette, and the positive mutant ratio is only 2% | Targeting efficiency depends on the homologous recombination efficiency of host | Sun et al. 2017 |
|  | *R. toruloides* ATCC 10657 | 1. The positive ratio for targeted knockout of KU70 and CAR2 genes were 5.7% and 10.5%, respectively  2. Failed to obtain correct strain with STE20 or URA3 genes knockout | The targeted knockout rate of wild type depends on the length (>250 bp) of the homology arms | Koh et al. 2014 |
|  | *R. toruloides* ATCC 10657 △ku70 | The positive knockout ratio of STE20, URA3, and CAR2 increased to 2.1%, 95.8%, and 75.3%, respectively | 1. The targeted knockout rate of the KU70 deficient strain depends on the length (> 100 bp) of the homology arms;  2. Positive ratio of the KU70 deficient strain is up to 43-fold higher than that of wild type | Koh et al. 2014 |
| LiAc/PEG-mediated chemical transformation (random insertion) | *R. toruloides* DMKU3-TK16 | 1. DNA fragment harboring a resistance marker cassette can be transformed;  2. Transformation efficiency is 25 transformants/μg DNA. | 1. The vector is a linear DNA fragment and randomly inserted into a chromosome;  2. The transformation step is simple and time-saving.  3. The transformation efficiency is low and affected by various operational factors. | Tsai et al. 2017 |
| Electrotransformation | *R. gracilis* ATCC 26217 | 1. ura3 gene is replaced by the transformed DNA fragment harboring a resistance marker cassette;  2. Transformation efficiency is 40 CFU/μg DNA. | 1. Linear DNA fragment preferred;  2. Relatively simple and time-saving transformation procedure. | Takahashi S et al. 2014 |
|  | *R. toruloides* NP11  *R. toruloides* NP5-2  *R. toruloides* Y4 | 1. Transformation with DNA fragment harboring a resistance marker cassette;  2. Competent cells are more efficient in transformation after pretreatment with DTT and LiAc;  3. Optimal transformation efficiency is above 1000 CFU/μg DNA |  | Liu et al. 2017 |

Table S3 Genetic parts used in *Rhodosporidium* species.

| Genes | Function | Gene resources | Promoter/Terminator | Host | References |
| --- | --- | --- | --- | --- | --- |
| *hpt-3* | Resistant to hygromycin | Plasmid pMF2-3c | P*_GPD1_*/T*_35S_*^1^, P*_GPD1_*/T*_SV40_*^2^ | *R. toruloides* ATCC 10657 | Liu et al. 2013; Koh et al. 2014; Liu et al. 2015 |
| *hygB* | Resistant to hygromycin | Codon-optimized gene, KF826486 | P*_PGK_*/T*_NOS_*, P*_GPD_*/T*_NOS_*, P*_GPD_*/T*_hsp_*, P*_PGK_*/T*_hsp_*, P*_PGI_*/T*_NOS_*, P*_FBA_*/T*_NOS_*, P*_TPI_*/T*_NOS_* | *R. toruloides* NP11 | Lin et al. 2014; Wang et al. 2016 |
| *ble* | Resistant to bleomycin | Plasmid pPICZaA | P*_ura3_*/T*_ura3_*, P*_ura5_*/T*_ura5_*, P*_GPD_*/T*_NOS_*, P*_INO_*/T*_ino_*, P*_LRO_*/T*_LRO_*, P*_DGA_*/T*_DGA_* | *R. toruloides* NP11 | Lin et al. 2014; |
| *ble* | Resistant to zeocin | Plasmid pPlCZA | P*_ura3_*/T*_ura3_* | *R. gracilis* ATCC 26217 | Takahashi et al. 2014 |
| *nat* | Resistant to nourseothricin | Plasmid pNGR1 | P*_PGK_*/T*_NOS_* | *R. toruloides* NP11 | Lin et al. 2014; |
| *G418* | Resistant to geneticin | Codon-optimized gene | P*_PGK47_*/T*_35S_* | *R. toruloides* CECT 13085, *R. toruloides* CBS 14 | Fillet et al. 2015; Fillet et al. 2017; Johns et al. 2016 |
| *URA3* | Uracil auxotrophic marker | *R. toruloides* NP11 | P*_ura3_*/T*_ura3_* | *R. toruloides* NP11 | Yang et al. 2008 |
| *LEU2* | Leucine auxotrophic marker | *S. cerevisiae* | P*_leu2_*/T*_leu2_* | *R. toruloides* MS7013 | Tully and Gilbert, 1985 |
| *LEU2* | Leucine auxotrophic marker | *R. toruloides* CBS 14 | **P*_ICL1_***/T*_35S_*, **P*_NAR1_***/T*_35S_*, **P*_MET16_***/T*_35S_*, **P*_CTR3_***/T*_35S_* | *R. toruloides* CBS 14 | Johns et al. 2016 |
| *eGFP* | Coding enhanced green fluorescent protein | Codon-optimized gene | P*_gpd_*/T*_nos_* | *R. toruloides* ATCC 10657 | Liu et al. 2013 |
| *eGFP* | Coding enhanced green fluorescent protein | Codon-optimized gene | **P*_ICL1_***/T*_35S_*, **P*_NAR1_***/T*_35S_*, **P*_MET16_***/T*_35S_*, **P*_CTR3_***/T*_35S_* | *R. toruloides* CBS 14 | Johns et al. 2016 |
| *GFP* | Coding green fluorescent protein | Codon-optimized gene, MF405194 | P*_LAD_*/T*_nos_*, P*_XYL1_*/T*_nos_*, P*_PGK47_*/T*_nos_*, P*_TEF1_*/T*_nos_*, P*_GPD1_*/T*_nos_* | *R. toruloides* CECT 13085 | Diaz et al. 2018 |
| *Luc* | Coding luciferase | Codon-optimized gene of luciferase, ACH53166.1 | **P*_DAO1_***/T*_35S_*, P*_GPD1_*/T*_35S_*, P*_ACL1_*/T*_35S_*, P*_FAS1_*/T*_35S_*, P*_DUR1_*/T*_35S_*, P*_FAT1_*/T*_35S_*, P_LDP1_/T*_35S_* | *R. toruloides* ATCC 10657 | Liu et al. 2015 |
| *mRuby2* | Coding fluorescent protein mRUBY2 | Synthetic gene | 20 endogenous constitutive promoters (12 monodirectional and 8 bidirectional) | *R. toruloides* IFO 0880 ΔKu70 | Nora et al. 2019 |

Bold font, inducible promoters; ^1^, derived from *Cauliflower mosaic virus*; ^2^, derived from *Agrobacterium tumefaciens*.

Table S4 Summary of strain engineering performed in *Rhodosporidium* species.

| Strain engineering | Host strains | Strategy | Description | References |
| --- | --- | --- | --- | --- |
| Product export/secretion engineering | *R. toruloides* CBS 5490 | Integration of the gene encoding a membrane transporter, pleiotropic drug resistance (Pdr) 10 from *S. cerevisiae* | 1. Carotenoid efflux efficiency increased by 5-fold;  2. Concurrent carotenoids/intracellular lipids separation;  3. Carotenoid yield increase from 1.9 to 2.9 μg/mg. | Lee et al. 2016 |
| Lipid production promotion | *R. toruloides* NP11 | Developing a new acetyl-CoA synthesis pathway via integration of the gene encoding phosphotransacetylase from *Bacillus subtilis* | Cell mass, lipid yield and lipid productivity enhanced by 26%, 11% and 54%, respectively | Yang et al 2018 |
|  | *R. toruloides* CECT 13085 | Random integration of genes encoding endogenous DGA1 and SCD1 via ATMT | 1. Lipid yield increased by 13.3%;  2. Lipid titer reached 39.5 g/L from undetoxified lignocellulosic hydrolysates. | Diaz et al. 2018 |
|  | *R. toruloides* IFO 0880 | Random integration of genes encoding endogenous ACC1 and DGA1 via ATMT | 1. Lipid titer increased by 74.6% to 24.8 g/L;  2. Lipid titer increased by 2.49-fold to 62.8 g/L in a fed-batch culture. | Zhang et al. 2016 |
|  | *R. toruloides* IFO 0880 | Random integration of genes encoding endogenous ACC1, DGA1 and SCD1 genes via ATMT | 1. Lipid titer increased by 85.1% to 27.4 g/L;  2. Lipid titer increased by 3.97-fold to 89.4 g/L in a fed-batch culture. | Zhang et al. 2016 |
|  | *R. toruloides* NBRC 8766 | UV mutagenesis plus orientation screening | 1. Lipid output and productivity increased significantly;  2. Transcriptional analysis revealed dramatic up-regulation of IDP1 and ME1 gene. | Yamada et al. 2017 |
|  | *R. toruloides* NP11 | Atmospheric and room temperature plasma (ARTP) and chemical mutagenesis | Lipid and carotenoid production improved simultaneously | Zhang et al. 2016 |
|  | *R. toruloides* AS 2.1389 | Random integration of the gene encoding endogenous malic enzyme via ATMT | Lipid content and yield increased by 84% and 91%, respectively, under phosphorus-limited conditions. |  |
| Diversity exploration of fatty acids derived- product | *R. toruloides* AS 2.1389 | Random integration of the genes encoding Δ12-fatty acid desaturase from *Fusarium verticillioides* | Linoleic acid content increased by 5-fold to a final linoleic acid titer of 1.3 g/L under flask culture conditions | Wang et al. 2016 |
|  | *R. toruloides* CECT 13085 | Random integration of genes encoding Δ12 desaturase and ω-3 desaturase with in-frame deletion of aldehyde dehydrogenase via ATMT | α-Linolenic acid content reached ~ 49% of total fatty acids | Fillet et al. 2016 |
|  | *R. toruloides* CECT 13085 | Random integration of genes encoding 3-ketoacyl-CoA synthase from different plants | Produced very long-chain fatty acids erucic acid and nervonic acid at final titers of 5.8 g/L and 7.9 g/L, respectively | Fillet et al. 2017 |
|  | *R. toruloides* CECT 13085 | Random integration of the gene encoding fatty acyl-CoA reductase via ATMT | Long chain (C16-C18) fatty alcohols were secreted extracellularly with final titers up to 8.0 g/L | Fillet et al. 2015 |
|  | *R. toruloides* NP11  *R. toruloides* TK16 | Random integration of genes encoding ScOle1 and the homologue RtΔ9Fad via LiAc/PEG-mediated chemical transformation | Produced lipids with 5-fold more oleic acid content | Tsai et al. 2019 |
| Metabolic engineering for production of terpenoids | *R. toruloides* IFO 0880 | Random integration of genes encoding terpene synthases via ATMT | Produced 1,8‑cineole at up to 34.6 mg/L from biomass hydrolysates | Zhuang et al. 2019 |
|  | *R. toruloides* IFO 0880 | Random integration of genes encoding bisabolene synthase and amorphadiene synthase via ATMT | 1. Produced bisabolene at 680 mg/L on the alkaline corn stover hydrolysates;  2. Bisabolene titers improved to 2.2 g/L in 20-L bioreactor, using separation-free biomass hydrolysates as substrate. | Yaegashi et al. 2017; Sundstrom et al. 2018; Pimienta et al. 2019 |
| Metabolic engineering for non-ribosomal peptides production | *R. toruloides* IFO 0880 | Random integration of genes encoding *BpsA* and *sfp* via ATMT | Produced indigoidine at up to 2.9 ± 0.8 g/L using a sorghum biomass hydrolysates in a batch process and 86.3 ± 7.4 g/L using glucose in a high-gravity fed-batch process | Wehrs et al. 2019 |
| Stress-resistance improvement | *R. toruloides* ACCC 20341 | Atmospheric and room temperature plasma mutagenesis | 1. Obtain mutant strain with higher tolerance to biomass hydrolysates inhibitor;  2. Both cell mass and lipid content increased by over 10%. | Qi et al. 2014 |

**References**

Bommareddy RR, Sabra W, Maheshwari G *et al.* Metabolic network analysis and experimental study of lipid production in *Rhodosporidium toruloides* grown on single and mixed substrates. *Microb Cell Fact* 2015;**14**:36.

Capusoni C, Rodighiero V, Cucchetti D *et al.* Characterization of lipid accumulation and lipidome analysis in the oleaginous yeasts *Rhodosporidium azoricum* and *Trichosporon oleaginosus*. *Bioresour Technol* 2017;**238**:281-9.

Castaneda MT, Nunez S, Garelli F *et al*. Comprehensive analysis of a metabolic model for lipid production in *Rhodosporidium toruloides*. *J Biotechnol* 2018;**280**:11-8.

Deligios M, Fraumene C, Abbondio M *et al.* Draft genome sequence of *Rhodotorula mucilaginosa*, an emergent opportunistic pathogen. *Genome Announc* 2015;**3**: e00201-15.

Diaz T, Fillet S, Campoy S *et al*. Combining evolutionary and metabolic engineering in *Rhodosporidium toruloides* for lipid production with non-detoxified wheat straw hydrolysates. *Appl Microbiol Biotechnol* 2018;**102**:3287-300.

Fillet S, Gibert J, Suarez B *et al.* Fatty alcohols production by oleaginous yeast. *J Ind Microb Biot* 2015;**42**:1463-72.

Fillet S, Ronchel C, Callejo C *et al*. Engineering Rhodosporidium toruloides for the production of very long-chain monounsaturated fatty acid-rich oils. *Appl Microbiol Biotechnol* 2017;**101**:7271-80.

Fillet SC, GonzalezBS, Barreno MDCR *et al.* Production of microbial oils with an elevated oleic acid content. *WO2016185073A1* 2016.

Firrincieli A, Otillar R, Salamov A *et al.* Genome sequence of the plant growth promoting endophytic yeast *Rhodotorula graminis* WP1. *Front Microbiol* 2015;**6**:978.

Goordial J, Raymond-Bouchard I, Riley R *et al*. Improved high-quality draft genome sequence of the eurypsychrophile *Rhodotorula sp*. JG1b, isolated from permafrost in the hyperarid upper-elevation mcMurdo dry valleys, Antarctica. *Genome Announc* 2016;**4**: e00069-16.

Hu J, Ji L. Draft genome sequences of Rhodosporidium toruloides strains ATCC 10788 and ATCC 10657 with compatible mating types. *Genome Announc* 2016;**4**:e00098-00016.

Johns AMB, Love J, Aves SJ *et al*. Four inducible promoters for controlled gene expression in the oleaginous yeast *Rhodotorula toruloides*. *Front Microbiol* 2016;**7**:1666.

Koh CMJ, Liu YB, Moehninsi *et al.* Molecular characterization of KU70 and KU80 homologues and exploitation of a KU70-deficient mutant for improving gene deletion frequency in *Rhodosporidium toruloides*. *BMC Microbiol* 2014;**14**:50.

Kumar S, Kushwaha H, Bachhawat AK *et al.* Genome sequence of the oleaginous red yeast *Rhodosporidium toruloides* MTCC 457. *Eukaryo Cell* 2012;**11**:1083-4.

Lee JJL, Chen LW, Cao B *et al*. Engineering *Rhodosporidium toruloides* with a membrane transporter facilitates production and separation of carotenoids and lipids in a bi-phasic culture. *Appl Microbiol Biotechnol* 2016;**100**:869-77.

Lee JJL, Chen LW, Shi JH *et al.* Metabolomic profiling of *Rhodosporidium toruloides* grown on glycerol for carotenoid production during different growth phases. J. Agric Food Chem 2014;**62**:10203-9.

Lin X, Wang Y, Zhang S *et al.* Functional integration of multiple genes into the genome of the oleaginous yeast *Rhodosporidium toruloides.* *FEMS Yeast Res* 2014;**14**:547-55.

Liu H, Jiao X, Wang Y *et al*. Fast and efficient genetic transformation of oleaginous yeast *Rhodosporidium toruloides* by using electroporation. *FEMS Yeast Res* 2017;**17**: fox017.

Liu H, Zhao X, Wang F *et al.* Comparative proteomic analysis of *Rhodosporidium toruloides* during lipid accumulation. *Yeast* 2009;**26**:553-66.

Liu YB, Koh CMJ, Ngoh ST *et al.* Engineering an efficient and tight D-amino acid-inducible gene expression system in *Rhodosporidium/Rhodotorula* species. Microb Cell Fact 2015;**14**:170.

Liu YB, Koh CMJ, Sun LH *et al*. Characterization of glyceraldehyde-3-phosphate dehydrogenase gene RtGPD1 and development of genetic transformation method by dominant selection in oleaginous yeast *Rhodosporidium toruloides*. *Appl Microbiol Biotechnol* 2013;**97**:719-29.

Morin N, Calcas X, Devillers H *et al*. Draft genome sequence of *Rhodosporidium toruloides* CECT1137, an oleaginous yeast of biotechnological interest. *Genome Announc* 2014;**2**:e00641-00614.

Nora LC, Wehrs M, Kim J, Cheng J-F, Tarver A, Simmons BA, et al. A toolset of constitutive promoters for metabolic engineering of *Rhodosporidium toruloides*. Microb Cell Fact. 2019;**18**:117.

Paul D, Magbanua Z, Arick M *et al.* Genome sequence of the oleaginous yeast *Rhodotorula glutinis* ATCC 204091. *Genome Announc* 2014;**2**:e00046-00014.

Pimienta JAP, Papa G, Rodrigue A *et al.* Pilot-scale hydrothermal pretreatment and optimized saccharification enables bisabolene production from multiple feedstock. *Green Chem* 2019;**21**: 3152-64.

Qi F, Kitahara Y, Wang ZT *et al*. Novel mutant strains of *Rhodosporidium toruloides* by plasma mutagenesis approach and their tolerance for inhibitors in lignocellulosic hydrolyzate. ‎*J Chem Technol Biotechnol* 2014;**89**:735-42.

Qi F, Zhao XB, Kitahara Y *et al.* Integrative transcriptomic and proteomic analysis of the mutant lignocellulosic hydrolyzate-tolerant *Rhodosporidium toruloides*. *Eng Life Sci* 2017;**17**:249-61.

Shi J, Feng H, Lee J *et al*. Comparative proteomics profile of lipid-cumulating oleaginous yeast: an iTRAQ-coupled 2-D LC-MS/MS analysis. *PLoS One*. 2013;**8**:e85532.

Sun W, Yang X, Wang X *et al.* Homologous gene targeting of a carotenoids biosynthetic gene in *Rhodosporidium toruloides* by Agrobacterium-mediated transformation. *Biotechnol Lett* 2017, **39**:1001-7.

Sundstrom E, Yaegashi J, Yan J *et al.* Demonstrating a separation-free process coupling ionic liquid pretreatment, saccharification, and fermentation with *Rhodosporidium toruloides* to produce advanced biofuels. *Green Chem* 2018;**20**:2870-9.

Takahashi S, Okada H, Abe K *et al.* Genetic transformation of the yeast *Rhodotorula gracilis* ATCC 26217 by electroporation. *Appl Biochem Microbiol* 2014;**50**:624-8.

Tiukova IA, Brandenburg J, Blomqvist J *et al.* Proteome analysis of xylose metabolism in *Rhodotorula toruloides* during lipid production. *Biotechnol Biofuels* 2019a;**12**:137.

Tiukova IA, Prigent S, Nielsen J *et al.* Genome‐scale model of *Rhodotorula toruloides* metabolism. *Biotechnol Bioeng* 2019b;**116**: 3396–408.

Tkavc R, Matrosova VY, Grichenko OE *et al*. Prospects for fungal bioremediation of acidic radioactive waste sites: Characterization and genome sequence of *Rhodotorula taiwanensis* MD1149. *Front Microbiol* 2018;**8**:2528.

Tran TN, Ngo D-H, Nguyen, NT *et al*. Draft genome sequence data of *Rhodosporidium toruloides* VN1, a strain capable of producing natural astaxanthin. *Data Brief* 2019;**26**:104443.

Tsai YY, Ohashi T, Kanazawa T *et al.* Development of a sufficient and effective procedure for transformation of an oleaginous yeast, *Rhodosporidium toruloides* DMKU3-TK16. *Curr Genet* 2017;**63**:359-71.

Tsai YY, Ohashi T, Wu CC *et al.* Delta-9 fatty acid desaturase overexpression enhanced lipid production and oleic acid content in *Rhodosporidium toruloides* for preferable yeast lipid production. *J Biosci Bioeng* 2019;**127**:430-40.

Tully M, Gilbert HJ. Transformation of Rhodosporidium toruloides. *Gene* 1985;**36**:235-40.

Wang Y, Zhang S, Potter M *et al.* Overexpression of Δ12-fatty acid desaturase in the oleaginous yeast Rhodosporidium toruloides for production of linoleic acid-rich lipids. *Appl Biochem Biotechnol* 2016;**180**:1497-507.

Wang Y, Zhang S, Zhu Z *et al.* Systems analysis of phosphate-limitation-induced lipid accumulation by the oleaginous yeast *Rhodosporidium toruloides*. *Biotechnol Biofuels* 2018;**11**:148.

Wang Y, Lin X, Zhang S *et al*. Cloning and evaluation of different constitutive promoters in the oleaginous yeast *Rhodosporidium toruloides*. *Yeast* 2016;**33**:99-106.

Wehrs M, Gladden JM，Liu Y *et al.* Sustainable bioproduction of the blue pigment indigoidine: Expanding the range of heterologous products in *R. toruloides* to include non-ribosomal peptides. *Green Chem* 2019;**21**:3394.

Yaegashi J, Kirby J, Ito M *et al*. *Rhodosporidium toruloides*: a new platform organism for conversion of lignocellulose into terpene biofuels and bioproducts. *Biotechnol Biofuels* 2017;**10:**241.

Yamada R, Kashihara T, Ogino H. Improvement of lipid production by the oleaginous yeast *Rhodosporidium toruloides* through UV mutagenesis. *World J Microb Biot* 2017;**33**:99.

Yang F, Zhang S, Tang W *et al.* Identification of the orotidine‐5′‐monophosphate decarboxylase gene of the oleaginous yeast *Rhodosporidium toruloides*. *Yeast* 2008;**25**:623-30.

Yang X, Sun W, Shen H *et al.* Expression of phosphotransacetylase in *Rhodosporidium toruloides* leading to improved cell growth and lipid production. *RSC Adv* 2018;**8**:24673-8.

Zhang C, Shen H, Zhang X *et al.* Combined mutagenesis of *Rhodosporidium toruloides* for improved production of carotenoids and lipids. *Biotechnol lett* 2016;**38**:1733-8.

Zhang S, Skerker JM, Rutter CD *et al*. Engineering *Rhodosporidium toruloides* for increased lipid production. *Biotechnol Bioeng* 2016;**113**:1056-66.

Zhang S, Ito M, Skerker JM *et al.* Metabolic engineering of the oleaginous yeast *Rhodosporidium toruloides* IFO0880 for lipid overproduction during high-density fermentation. *Appl Microbiol Biotechnol* 2016;**100**:9393-405.

Zhu Z, Zhang S, Liu H *et al.* A multi-omic map of the lipid-producing yeast *Rhodosporidium toruloides*. *Nat Commun* 2012;**3**:1112.

Zhu Z, Ding Y, Gong Z *et al.* Dynamics of the  lipid droplet proteome of the oleaginous yeast *Rhodosporidium toruloides*. *Eukaryot Cell* 2015;**14**:252-64.

Zhuang X, Kilian O, Monroe E *et al*. Monoterpene production by the carotenogenic yeast *Rhodosporidium toruloides*. *Microb Cell Fact* 2019;**18**:54.
